# The wide world of technological telerehabilitation for pediatric neurologic and neurodevelopmental disorders – a systematic review

**DOI:** 10.3389/fpubh.2024.1295273

**Published:** 2024-04-17

**Authors:** Benedetta Del Lucchese, Stefano Parravicini, Silvia Filogna, Gloria Mangani, Elena Beani, Maria Chiara Di Lieto, Alessandra Bardoni, Marta Bertamino, Marta Papini, Chiara Tacchino, Francesca Fedeli, Giovanni Cioni, Giuseppina Sgandurra, the Italian Neuroscience and Neurorehabilitation Network, Maria Teresa Arnoldi, Francesca Baglio, Veronica Barzacchi, Maria Teresa Bassi, Angela Berardinelli, Clara Bombonato, Renato Borgatti, Rocco Salvatore Calabrò, Ilaria Cardillo, Enrico Castelli, Anna Cavallini, Beatrice Ceragioli, Antonella Cersosimo, Claudia Condoluci, Claudia Corti, Gabriella Di Girolamo, Valentina Di Giusto, Maurizio Elia, Martina Favetta, Carolina Ferrante, Raffaele Ferri, Valeria Ghione, Michela Goffredo, Patrizia Lugari, Carlotta Maria Manzia, Giada Martini, Elisa Matteucci, Valentina Menici, Paolo Moretti, Emanuela Pagliano, Martina Giorgia Perinelli, Maurizio Petrarca, Geraldina Poggi, Francesca Pulvirenti, Marta Rizzo, Giada Sgherri, Sandra Strazzer, Pasquale Striano, Cristina Tassorelli, Federica Vannetti, Marta Viganò

**Affiliations:** ^1^Department of Developmental Neuroscience, IRCCS Fondazione Stella Maris Foundation, Pisa, Italy; ^2^Department of Brain and Behavioral Sciences, University of Pavia, Pavia, Italy; ^3^Pediatric Neuroscience Center, IRCCS Mondino Foundation, Pavia, Italy; ^4^Department of Clinical and Experimental Medicine, University of Pisa, Pisa, Italy; ^5^Scientific Institute, IRCCS E. Medea, Lecco, Italy; ^6^Physical Medicine and Rehabilitation Unit, IRCCS Istituto Giannina Gaslini, Genova, Italy; ^7^Fightthestroke Foundation, Milan, Italy

**Keywords:** technologies, telerehabilitation, pediatric, neurodevelopmental disorders, neurological disorders, children, home-based

## Abstract

**Introduction:**

The use of Information and Communication Technology (ICT) for assessing and treating cognitive and motor disorders is promoting home-based telerehabilitation. This approach involves ongoing monitoring within a motivating context to help patients generalize their skills. It can also reduce healthcare costs and geographic barriers by minimizing hospitalization. This systematic review focuses on investigating key aspects of telerehabilitation protocols for children with neurodevelopmental or neurological disorders, including technology used, outcomes, caregiver involvement, and dosage, to guide clinical practice and future research.

**Method:**

This systematic review adhered to PRISMA guidelines and was registered in PROSPERO. The PICO framework was followed to define the search strategy for technology-based telerehabilitation interventions targeting the pediatric population (aged 0–18) with neurological or neurodevelopmental disorders. The search encompassed Medline/PubMed, EMBASE, and Web of Science databases. Independent reviewers were responsible for selecting relevant papers and extracting data, while data harmonization and analysis were conducted centrally.

**Results:**

A heterogeneous and evolving situation emerged from our data. Our findings reported that most of the technologies adopted for telerehabilitation are commercial devices; however, research prototypes and clinical software were also employed with a high potential for personalization and treatment efficacy. The efficacy of these protocols on health or health-related domains was also explored by categorizing the outcome measures according to the International Classification of Functioning, Disability, and Health (ICF). Most studies targeted motor and neuropsychological functions, while only a minority of papers explored language or multi-domain protocols. Finally, although caregivers were rarely the direct target of intervention, their role was diffusely highlighted as a critical element of the home-based rehabilitation setting.

**Discussion:**

This systematic review offers insights into the integration of technological devices into telerehabilitation programs for pediatric neurologic and neurodevelopmental disorders. It highlights factors contributing to the effectiveness of these interventions and suggests the need for further development, particularly in creating dynamic and multi-domain rehabilitation protocols. Additionally, it emphasizes the importance of promoting home-based and family-centered care, which could involve caregivers more actively in the treatment, potentially leading to improved clinical outcomes for children with neurological or neurodevelopmental conditions.

**Systematic review registration:**

PROSPERO (CRD42020210663).

## 1 Introduction

### 1.1 Neurodevelopmental disabilities

Neurodevelopmental disorders, encompassing conditions such as attention deficit and hyperactivity disorder (ADHD), autism spectrum disorder (ASD), specific learning disabilities (SLD), developmental coordination disorder (DCD), and intellectual disability (ID), collectively represent a relevant nosographic group in pediatric age. These disorders, along with some neurological diseases (e.g., cerebral palsy), interfere with typical neurodevelopment, and they are a frequent cause of significant disability in pediatric patients. Motor, neuropsychological, and language impairments are possibly part of the clinical picture in these diseases, impacting the daily functioning and quality of life. The complexity of these conditions raises the need for comprehensive rehabilitation strategies addressing the organicity of the process of neurodevelopment. Motor impairments often lead to challenges in mobility and coordination, while neuropsychological and language deficits interfere with the acquisition of cognitive and communicative skills.

Long-term rehabilitation (or re-habilitation, if we adopt the perspective of sustaining the acquisition of a developing skill other than “restoring” a lost one) associated with an ecological rehabilitation approach, integrating therapies within the patient’s familiar environment, is crucial for effective intervention. Thus, telerehabilitation emerged as a promising field to enhance treatment efficacy and compliance and reduce the burden on patients and their families. Tele-rehabilitation not only provides accessibility to therapeutic interventions but also facilitates continuous monitoring and adaptation of rehabilitation programs to meet evolving needs. Moreover, implementing innovative technologies in rehabilitation can merge these advantages into a holistic and patient-centered approach.

### 1.2 Telerehabilitation: main features and conveniences

The recent development and availability of Internet and Communication Technologies (ICTs) have fostered the possibility of applying technology-based solutions to provide health services both during hospitalization and after discharge from the hospital (1), also for children with neurodevelopmental disabilities or neurological conditions. The World Health Organization (WHO) defines telehealth as the “delivery of health care services, where patients and providers are separated by distance. Telehealth uses information communication technology for the exchange of information for the diagnosis and treatment of diseases and injuries, research and evaluation, and for the continuing education of health professionals” (2). Over the past 3 years, an increasing interest in developing and applying user-friendly technological systems has become even more highlighted. The unexpected COVID-19 pandemic has driven the introduction of security measures and restrictions to preserve public health, substantially impacting clinical activities and rehabilitation services for neurodevelopmental disabilities (3). Such abrupt interruption or the reduction of access to non-emergency face-to-face diagnostic and rehabilitative procedures have had adverse short- and long-term consequences for patients with neuropsychological and motor disorders and their caregivers (4), thus pushing forward the uptake of telehealth, as the only way to continue the clinical practice, with promising results (5–8). Among different applications of the technologies in clinical practice (assessment, consultation, monitoring), ICTs have become a valuable option for rehabilitation, enabling timely and tailored therapeutic interventions (9).

Telerehabilitation programs foster access to rehabilitative services and permit the delivery of a wide range of neuropsychological, motor, speech and communication interventions, even for patients unable to frequently attend a clinical institution (distance from the hospital, parental work employment, etc.), by overcoming geographic barriers. In this scenario, new technologies guarantee significant time- and cost-saving, shortening hospitalization and delivering the rehabilitative process at home, in a more ecological context therefore enforcing the generalization of the achieved competences.

Another great advantage provided by using innovative technologies in clinical practice to foster therapies tailored to patient’s needs concerns both the possibility of collecting comprehensive and accurate quantitative data, thus supporting a better intervention monitoring, and of offering multi domain activities, also integrating peripheral devices (i.e., sensors). Using innovative technologies in clinical practice also give the possibility to propose neuropsychological and motor activities in a playful and motivating context, thus enhancing participation and enjoyment, especially for the pediatric population, while maintaining high levels of efficiency (10, 11). Such telerehabilitation pathways allow to increase dosage and intensity of the intervention (12) and ensure caregivers’ involvement in the rehabilitation process. The parental role in rehabilitation interventions is described as the set of tasks or responsibilities attributed to caregivers during the intervention (13), placed on a continuum from a passive to an active involvement (14), in passive roles, parents comply with interventions driven by the expert professional, ensuring children’s attendance at rehabilitative sessions and supporting their enthusiasm and motivation to participate; conversely, in more active roles, parents are involved as “leaders,” bringing a personal contribution to the intervention sessions and also collaborating in the decision-making steps. Both intensity and parental involvement are described as features supportive of the rehabilitation effectiveness in children with neurodevelopmental disorders, according to the main scientific literature and guidelines (15–17).

### 1.3 Internet and communication technologies classification

The progress of digital technologies (namely, associated with the use of computers, smartphones, the internet, and other digital devices and platforms) enabled the delivery of rehabilitation services via ICTs (18), by offering a vast world of possibilities, from interventions targeting separately motor, neuropsychological, speech and communication functions, to integrated rehabilitation pathways.

Despite the benefits offered by digital technologies and the increase in their use, strongly driven by the pandemic emergency, a standardized taxonomy able to classify the different existing digital technologies for telerehabilitation is still lacking.

In general, technologies can be classified based on their attributes and functionalities, depending on the context and the intended use. Likewise, this applies to digital health technologies; for instance, Camden and Silva (19) drafted a general classification of pediatric telehealth strategies able to offer personalized and home-based intervention based on the devices’ complexity from low-tech (e.g., phone calls and video/photo sharing), to high-tech solutions (e.g., specialized programs/serious games, virtual reality and sensors). A different example of digital technologies classification for motor rehabilitation in children has been proposed by The European Academy of Childhood Disability (EACD) (20). In this case, the classification involved three categories: (1) robotic devices and treadmills with body weight support systems; (2) virtual reality/gaming systems; (3) telehealth and phone/tablet apps. However, this classification does not consider many other evidence-based technologies that, to date, are utilized for rehabilitation interventions, mainly for cognitive functions.

Summarizing, although telerehabilitation yielded promising results in enhancing cognitive, motor, speech, and communication abilities, such intervention protocols still need to be routinely included in clinical practice. Several barriers exist to the adoption of ICTs technologies in pediatric intervention programs, both from the perspective of healthcare providers and families (e.g., limited access to the technology, cost implications, technological competency, privacy and data security concerns, lack of face-to-face interactions) (21). Furthermore, a critical gap exists in a systematic understanding and classification of the different ICTs employed in these interventions. Addressing these issues is therefore crucial for at least two reasons: (1) facilitating the successful implementation and acceptance of telerehabilitation into traditional pediatric care, consequently improving access to clinical services and outcomes for children with neurodevelopmental disabilities; (2) providing future research about technological telerehabilitation with useful elements to identify outcomes, compare different devices, and define intervention protocols.

This systematic review seeks therefore to bridge this gap by critically examining the wide world of technological devices for the intervention in children with neurodevelopmental disabilities. Moreover, by investigating the main features (e.g., type of adopted technology, functional domains identified as outcomes, caregiver involvement, dosage) supporting the effectiveness of telerehabilitation protocols, the review aims to provide valuable insights for guiding clinical practices, path further future studies, and support the use of innovative solutions for inclusive development. There is a general consensus that tele-rehabilitation cannot replace face-to-face intervention, but integrating technological devices proved to be feasible and effective in clinical practice, and could be a valuable contribution, leveraging the positive elements of this approach.

## 2 Method

### 2.1 Search strategy

The authors undertook a systematic search from four electronic databases Medline/PubMed, EMBASE, and Web of Science in February 2023, according to the Preferred Reporting Items for Systematic Reviews and Meta-Analyses (PRISMA) statement (22). Different combinations of keywords selected from analyzing recent scientific literature were used, particularly referring to four main clusters: “neurodevelopmental disabilities,” “children,” “telerehabilitation” and “home-based intervention.” Terms related to such constructs and definitions were also included (see Appendix 1 for the complete search string). In addition, the references of the included studies were also considered to identify additional eligible studies and to ensure comprehensive data collection. To exclude non-peer-reviewed studies, the authors included studies published in academic journals, reported in English, and available for full text. Considering that the development and the implementation of technological devices in telerehabilitation are relatively recent, articles published from 2000 were considered. The methodological quality of the included studies was assessed according to the National Health and Medical Research Council Evidence Hierarchy (NHMRC, 2009). This systematic review was registered on PROSPERO (CRD42020210663).

### 2.2 Inclusion and exclusion criteria

#### 2.2.1 Population

Studies were included when considering samples of children aged 0–18 years with motor, neuropsychological, cognitive, and speech-communication impairments due to neuropsychiatric conditions such as neurodevelopmental disorders including Specific Learning Disorders, Developmental Coordination Disorder, Language Disorder, Autism Spectrum Disorder, Attention Deficit Hyperactivity Disorder and Developmental Delay/Intellectual Disabilities (according to ICD 10 or DSM 5-IV TR) (23–25) genetic syndromes, prematurity, congenital or acquired brain lesions, and neuromuscular diseases.

#### 2.2.2 Interventions

The selected studies focused on telerehabilitation programs to improve motor, neuropsychological, cognitive, and speech-communication functions. Interventions had to be delivered entirely or partially (with almost a 50% percentage) in an ecological context such as home or school and through ICTs. According to the technologies classification reported in the following section, rehabilitation programs including virtual reality, active video gaming devices (i.e., Xbox, Kinect, Playstation), telemedicine and telemonitoring tools, computer-based programs and web-based platforms (i.e., CogMed RIDInet) were considered. Interventions should be monitored by health professional staff (such as psychologists, neuropsychiatrists, speech therapists, motor therapists, physiotherapists, and occupational therapists). Any frequency, intensity, and duration of the training was included. Moreover, the studies needed to have a pre-post treatment design or the presence of a control group (both active or waitlist).

##### 2.2.2.1 Classification of ICTs

Starting from the EACD classification, in this study we have defined a novel taxonomy for digital technologies to consider all the domains handled by the clinicians. Our proposal includes (i) Virtual reality and active video gaming devices (i.e., Xbox, Kinect, Playstation); (ii) Telemedicine and Telemonitoring devices; (iii) Computer-based program and web-based platform (i.e., CogMed RIDInet); (iv) other. Specifically, ‘other’ refers to purely robotic/treadmill systems that are difficult to transport and not entirely suitable for home-based treatment. This categorization manages to encompass all devices targeting purely motor, neuropsychological, or speech treatments but also integrated ones, thus combining motor and cognitive or cognitive and speech functions.

#### 2.2.3 Comparison

Both studies presenting a pre-post treatment evaluation and a comparison between experimental and control group—including alternative treatments or none (using a waiting list design)—were considered. Articles without a control group were also selected.

#### 2.2.4 Outcomes

Studies were included when quantitative measures of the efficacy of telerehabilitation interventions (i.e., standardized tests and scales administered to the child, clinicians/caregivers/self-report questionnaires, instrumental measurements) were adopted to assess neuropsychological, motor, cognitive, and speech-communication outcomes. Quality of life and daily life functioning were also considered as admissible outcomes.

The following exclusion criteria were considered (1): case reports, book chapters, conference abstracts, protocol studies, reviews (2); diagnostic or prognostic studies (3) participants aged >18 (4); samples with other medical, psychiatric or neurological conditions (5) interventions not based on ICTs (6); totally “clinic-based” interventions (7); interventions not primarily targeting neuropsychological, motor, speech and communication skills (8); quantitative outcome measures on the efficacy of the training not applied.

Feasibility studies were excluded unless they had pre- and post-treatment clinical measures as secondary outcomes.

### 2.3 Study selection process

**Figure 1:**
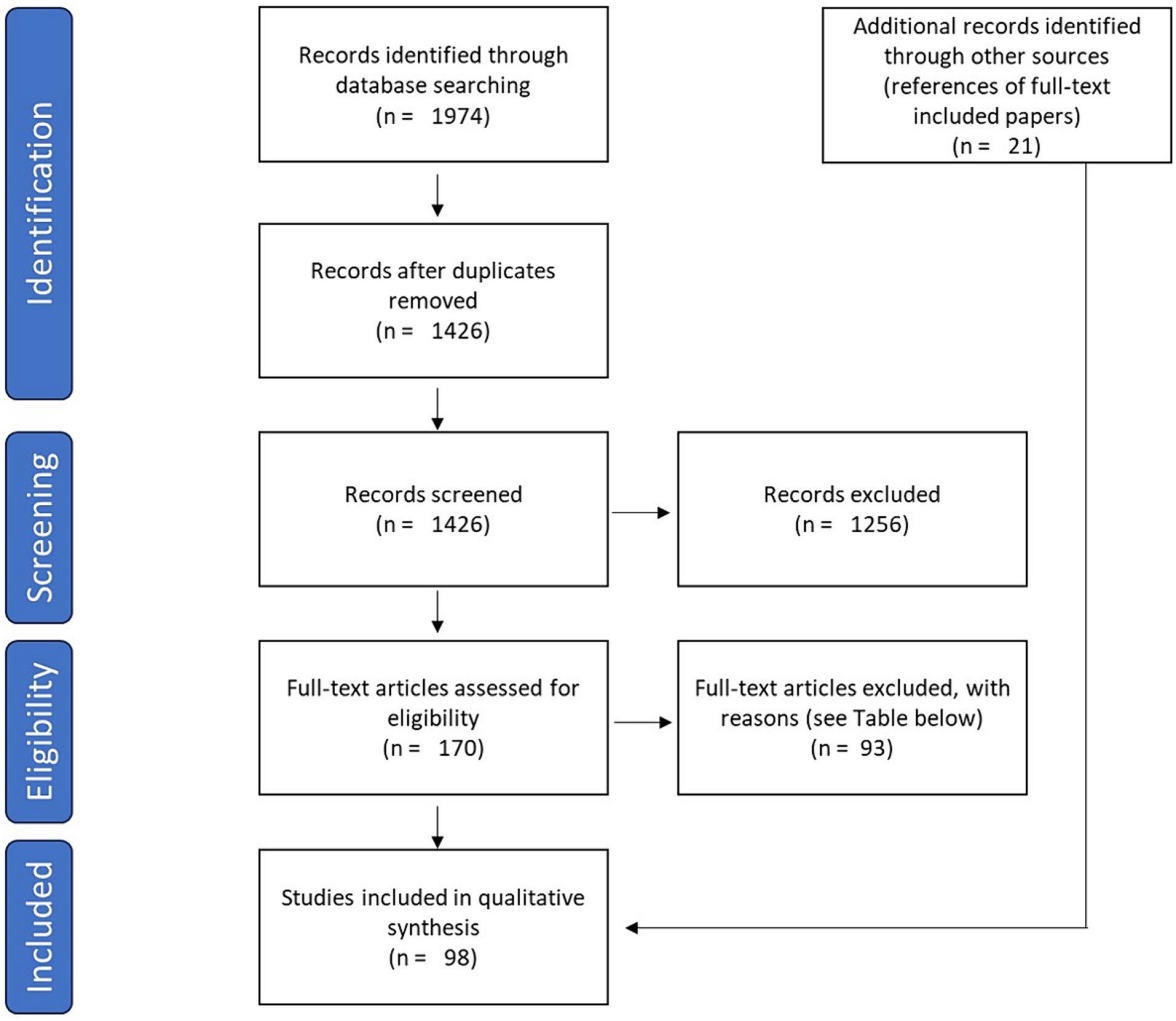
PRISMA Flow Diagram: the flow diagram represents the stages of the search strategy and the selection process of the articles included in the review, according to the Preferred Reporting Items for Systematic Reviews and Meta-Analyses (PRISMA) statement.

After automatically removing duplicates, pairs of independent authors screened the titles and abstracts of 1,427 articles. The resulting 170 articles were then further full text screened according to eligibility criteria, previously reassigning the set of papers to be reviewed by each pair of authors (compared to the title/abstract selection stage). In case of discrepancies, articles were discussed between the two reviewers to determine their inclusion or exclusion. If consensus could not be reached, a third reviewer was therefore consulted. References of the included studies were eventually reviewed to identify additional eligible studies. The process led to the selection of 98 papers that met the inclusion criteria. The overall process for selecting studies is shown in Figure 1 and Table 1.

**Table 1:** Reasons for full-text exclusion: the table provides an overview of the articles excluded per full-text examination, with details about the reasons for exclusion.

Reasons for exclusion of full-text assessed articles	
Exclusion criteria	*n*° of excluded papers
Reviews, case reports, book chapters, conference abstracts, protocol studies. [tag: article type]	29
Studies not including intervention based on technological devices (e.g., rehabilitation software, commercial videogames, sensors). [tag: technology]	26
Studies not applying quantitative outcome measures (assessed functions: motor function, neuropsychological functions, language, quality of life/daily life functioning). Feasibility studies not included [tag: outcome]	10
Studies not including totally or partially “home/school-based” interventions. [tag: intervention]	14
Studies including >18-year-old subjects or patients with non-neuropsychiatric disorders. [tag: population]	11
Studies about interventions not primarily targeting motor functions, neuropsychological functions or language. [tag: intervention target]	2
Studies on animals or about other disciplines. [tag: topic]	1
Total	**93**

### 2.4 Data extraction

For each paper included, the authors recorded in a dedicated database the following information: first author, title, year of publication, quality of the study (according to NHMRC Evidence Hierarchy), age range and diagnosis of the sample, study design, sample size, type of technologies used for intervention (see Introduction for the adopted classification), target functions of the rehabilitation program (motor, neuropsychological, speech/communication skills), direct target recipients of the interventions, intensity, frequency and duration of each treatment and outcome measures.

In particular, the framework proposed in a previously published review (14) has been adopted to classify the parental role in the rehabilitation process. Such classification includes eight different categories (Bringer, Supporter, Informer, Observer, Learner, Implementer, Adaptor, Collaborative Decision Maker), defining, in this order, a spectrum from passive to active responsibility.

Furthermore, considering the high heterogeneity of the studies, primary outcome measures were extracted and classified by two independent authors according to the International Classification of Functioning, Disability and Health-Children&Youth Version (ICF-CY) (26) domains, and core-set outcome measures that could be assigned to more than one ICF domain or core sets were classified considering the most prevalent one.

## 3 Results

The overall study selection process yielded 98 papers published between 2001 and 2023 (8, 27–121) (Table 2). The selected papers differed widely in all the considered parameters (i.e., study design, population, adopted technology, and outcome measures); thus, we analyzed the evidence grade, classifying them based on the NHMRC Levels (2009). None of the reviewed papers were included in Level I.

**Table 2:** Included papers: the details (authors, title, publication year) of the articles included in the qualitative analysis are reported in the table.

Author	Title	Publication year
Aarnoudse-Moens, et al.	Executive Function Computerized Training in Very Preterm-Born Children: A Pilot Study	2018
Alsaif, et al.	Effects of interactive games on motor performance in children with spastic cerebral palsy	2015
Anderson, et al.	Long-Term Academic Functioning following Cogmed Working Memory Training for Children Born Extremely Preterm: A Randomized Controlled Trial	2018
Bailey, et al.	A trial of online ABRACADABRA literacy instruction with supplementary parent-led shared book reading for children with autism	2022
Baque, et al.	Randomized controlled trial of web-based multimodal therapy for children with acquired brain injury to improve gross motor capacity and performance.	2017
Bearss, et al.	Feasibility of Parent Training via Telehealth for Children with Autism Spectrum Disorder and Disruptive Behavior: A Demonstration Pilot	2018
Benzing, et al.	The effect of exergaming on executive functions in children with ADHD: a randomized clinical trial	2019
Bikic, et al.	A double-blind randomized pilot trial comparing computerized cognitive exercises to Tetris in adolescents with attention-deficit/hyperactivity disorder	2017
Bilde, et al.	Individualized, home based interactive training of cerebral palsy children delivered through the internet	2011
Chacko, et al.	A randomized clinical trial of Cogmed Working Memory Training in school-age children with ADHD: A replication in a diverse sample using a control condition	2014
Chen, et al.	Efficacy of home-based virtual cycling training on bone mineral density in ambulatory children with cerebral palsy	2012
Chen, et al.	Efficacy of an integrated intervention with vocabulary and phonetic training for Mandarin-speaking children with developmental language disorders	2022
Chen, et al.	Muscle strength enhancement following home-based virtual cycling training in ambulatory children with cerebral palsy	2012
Chen, et al.	Home based tele assisted robotic rehabilitation of joint impairments in children with cerebral palsy	2014
Chiu, et al.	Upper limb training using Wii Sports Resort for children with hemiplegic cerebral palsy: a randomized, single-blind trial	2014
Chiu, et al.	Balance and mobility training at home using Wii Fit in children with Cerebral Palsy: a feasibility study	2018
Cohen, et al.	Effects of computer-based intervention through acoustically modified speech (Fast ForWord-FFW) in severe mixed receptive-expressive language impairment: outcomes from a randomized controlled trial	2005
Corti, et al.	Home based cognitive training in pediatric patients with acquired brain injury: preliminary results on efficacy of a randomized clinical trial	2020
Cristinziano, et al.	Telerehabilitation during COVID-19 lockdown and gross motor function in cerebral palsy: an observational study.	2022
Da Silva, et al.	Serious Game Platform as a Possibility for Home-Based Telerehabilitation for Individuals With Cerebral Palsy During COVID-19 Quarantine—A Cross-Sectional Pilot Study.	2021
Damiano, et al.	Task-Specific and Functional Effects of Speed-Focused Elliptical or Motor-Assisted Cycle Training in Children With Bilateral Cerebral Palsy: Randomized Clinical Trial.	2017
Davis, et al.	Proof-of-concept study of an at-home, engaging, digital intervention for pediatric ADHD.	2018
De Vries, et al.	Working memory and cognitive flexibility-training for children with an autism spectrum disorder: a randomized controlled trial	2015
Di Lieto, et al.	Adaptive Working Memory Training Can Improve Executive Functioning and Visuo-Spatial Skills in Children With Pre-term Spastic Diplegia	2021
Dovis, et al.	Improving Executive functioning in children with ADHD: Training multiple Executive Functions within the context of a computer Game. A randomized double-blind placebo controlled trial	2015
Egeland, et al.	Few Effects of Far Transfer of Working Memory Training in ADHD: A Randomized Controlled Trial	2013
Ferguson, et al.	The efficacy of two task-orientated interventions for children with Developmental Coordination Disorder: Neuromotor Task Training and Nintendo Wii Fit Training	2013
Garnett, et al.	Parent perceptions of a group telepractice communication intervention for autism	2022
Golomb, et al.	In home virtual reality videogame telerehabilitation in adolescents with hemiplegic cerebral palsy	2010
Goodwin, et al.	INTERSTAARS: attention training for infants with elevated likelihood of developing ADHD:a proof of concept randomized controlled trial	2021
Graucher, et al.	From Clinic Room to Zoom: Delivery of an Evidence-Based, Parent mediated Intervention in the Community Before and During the Pandemic	2022
Gray, et al.	Effects of a computerized working memory training program on working memory attention, and academics in adolescents with severe LD and comorbid ADHD: a randomized controlled trial	2012
Grunewaldt, et al.	Working Memory Training Improves Cognitive Function in VLBW Preschoolers	2013
Grunewaldt, et al.	Computerized working memory training has positive long-term effect in very low birthweight preschool children	2015
Hammond, et al.	An investigation of the impact of regular use of the Wii Fit to improve motor and psychosocial outcomes in children with movement difficulties: a pilot study	2012
Hardy, et al.	Computerized Working Memory Training for Children With Neurofibromatosis Type 1 (NF1): A Pilot Study	2021
Hessl, et al.	Cognitive training for children and adolescents with fragile X syndrome: a randomized controlled trial of Cogmed	2019
Howie, et al.	Understanding why an active video game intervention did not improve motor skill and physical activity in children with developmental coordination disorder: a quantity or quality issue?	2017
Howie, et al.	An active video game intervention does not improve physical activity and sedentary time of children at-risk for developmental coordination disorder: a crossover randomized trial	2015
Jaekel, et al.	Preterm children’s long-term academic performance after adaptive computerized training: an efficacy and process analysis of a randomized controlled trial	2021
Jirikowic, et al.	Virtual Sensorimotor Training for Balance: Pilot Study Results for Children With Fetal Alcohol Spectrum Disorders	2016
Johnstone, et al.	A pilot study of combined working memory and inhibition training for children with AD/HD	2009
Jouen, et al.	GOLiah (gaming open library for intervention in autism at home); a 6 month single blind matched controlled exploratory study	2017
Kassee, et al.	Home based nintendo wii training to improve upper limb function in children ages 7 to 12 with spastic hemiplegic cerebral palsy	2017
Kirk, et al.	Computerized attention training for children with intellectual and developmental disabilities: a randomized controlled trial	2016
Kirk, et al.	Impact of attention training on academic Achievement executive functioning and behavior: a randomized controlled trial	2017
Klingberg, et al.	Computerized Training of Working Memory in Children With ADHD—A Randomized, Controlled Trial	2005
Kollins, et al.	A novel digital intervention for actively reducing severity of pediatric ADHD (STARS-ADHD): a randomized controlled trial	2020
Kolobe, et al.	Robot Reinforcement and Error-Based Movement Learning in Infants With and Without Cerebral Palsy	2019
Lacava, et al.	Using assistive technology to teach emotion recognition to students with Asperger Syndrome	2007
Lanfranchi, et al.	Parent-based training of basic number skills in children with Down syndrome using an adaptive computer game	2021
Lee, et al.	Effects of working memory training on children born preterm	2016
Levac, et al.	Active Video Gaming for Children with Cerebral Palsy: Does a Clinic-Based Virtual Reality Component Offer an Additive Benefit? A Pilot Study	2018
Løhaugen, et al.	Computerized Working Memory Training Improves Function in Adolescents Born at Extremely Low Birth Weight	2010
Lorentzen, et al.	Twenty weeks of home-based interactive training of children with cerebral palsy improves functional abilities	2015
Luna-Oliva, et al.	Kinect Xbox 360 as a therapeutic modality for children with cerebral palsy in a school environment: a preliminary study	2013
Luo, et al.	A randomized controlled study of remote computerized cognitive, neurofeedback, and combined training in the treatment of children with attention-deficit/hyperactivity disorder	2022
MacIntosh, et al.	The design and evaluation of electromiography and inertial biofeedback in hand motor therapy gaming	2020
Magnan, et al.	Audio-visual training in children with reading disabilities	2006
Meguid, et al.	Influence of Covid 19 pandemic lockdown on a sample of Egyptian children with down syndrome	2022
Meyer, et al.	Computer-based inhibitory control training in children with Attention-Deficit/Hyperactivity Disorder (ADHD): Evidence for behavioral and neural impact	2020
Molinaro, et al.	Action Observation Treatment in a telerehabilitation setting	2020
Nuara, et al.	Efficacy of a home-based platform for child-to-child interaction on hand motor function in unilateral cerebral palsy	2019
Pascoe, et al.	Child motivation and family environment influence outcomes of working memory training in extremely preterm children	2019
Pecini, et al.	Telerehabilitation in developmental dyslexia: methods of implementation and expected results.	2018
Pecini, et al.	Training RAN or reading? A telerehabilitation study on developmental dyslexia	2019
Penev, et al.	A Mobile Game Platform for Improving Social Communication in Children with Autism: A Feasibility Study	2021
Piovesana, et al.	Randomized controlled trial of a web-based multi-modal therapy program for executive functioning in children and adolescents with unilateral cerebral palsy.	2017
Piovesana, et al.	A randomized controlled trial of a web-based multi-modal therapy program to improve executive functioning in children and adolescents with acquired brain injury	2017
Preston, et al.	A pilot single-blind multicenter randomized controlled trial to evaluate the potential benefits of computer-assisted arm rehabilitation gaming technology on the arm function of children with spastic cerebral palsy	2016
Preston, et al.	Feasibility of school-based computer-assisted robotic gaming technology for upper limb rehabilitation of children with cerebral palsy	2014
Prins, et al.	“Braingame Brian”: Toward an Executive Function Training Program with Game Elements for Children with ADHD and Cognitive Control Problems	2013
Pulina, et al.	Improving spatial simultaneous working memory in DOWN Syndrome: effect of a training program led by parents instead of an expert	2015
Ramstrand, et al.	Can balance in children with cerebral palsy improve through use of an activity promoting computer game?	2012
Re, et al.	Response to a Specific and Digitally Supported Training at Home for Students With Mathematical Difficulties	2020
Ronimus, et al.	Supporting struggling readers with digital game-based learning	2019
Sabel, et al.	Active video gaming improves body coordination in survivors of childhood brain tumors	2016
Sandlund, et al.	Training of goal directed arm movements with motion interactive video games in children with cerebral palsy—a kinematic evaluation	2014
Saniee, et al.	Developing set-shifting improvement tasks (SSIT) for children with high-functioning autism	2019
Sella, et al.	Training basic numerical skills in children with Down syndrome using the computerized game “the number race”	2021
Serrano-Gonzalez, et al.	Action Observation Training to Improve Activities of Daily Living and Manipulation Skills in Children with Acquired Brain Injury Secondary to an Oncologic Process: A Prospective Case Series Clinical Study	2022
Sgandurra, et al.	A pilot study on early home-based intervention through an intelligent baby gym (CareToy) in preterm infants	2016
Sgandurra, et al.	A randomized clinical trial in preterm infants on the effects of a home-based early intervention with the CareToy System	2017
Silver, et al.	Evaluation of a new computer intervention to teach people with autism or Asperger syndrome to recognize and predict emotions in others	2001
Simone, et al.	Computer-assisted rehabilitation of attention in pediatric multiple sclerosis and ADHD patients: a pilot trial	2018
Soderqvist, et al.	Computerized training of non-verbal reasoning and working memory in children with intellectual disability	2012
Steiner, et al.	Computer-Based Attention Training in the Schools for Children With Attention Deficit/Hyperactivity Disorder: A Preliminary Trial	2011
Straker, et al.	A crossover randomized and controlled trial of the impact of active video games on motor coordination and perceptions of physical ability in children at risk of Developmental Coordination Disorder	2015
Swenney, et al.	Randomized controlled trial comparing Parent Led Therapist Supervised Articulation Therapy (PLAT) with routine intervention for children with speech disorders associated with cleft palate.	2020
Tse, et al.	Teletherapy delivery of caregiver behavior training for children with attention-deficit hyperactivity disorder.	2015
Ura, et al.	Parent-Coaching Telehealth Intervention for Youth with Autism Spectrum Disorder: A Pilot Program	2021
Van der Molen, et al.	Effectiveness of a computerized working memory training in adolescents with mild to borderline intellectual disabilities	2010
van Dongen-Boomsma, et al.	Working memory training in young children with ADHD: a randomized placebo-controlled trial	2014
van Houdt, et al.	Executive function training in very preterm children: a randomized controlled trial	2020
Voss, et al.	Effect of Wearable Digital Intervention for Improving Socialization in Children With Autism Spectrum Disorder: A Randomized Clinical Trial	2019
Wang, et al.	Commercial exergaming in home-based pediatric constraint-induced therapy: a randomized trial	2021
Yoncheva, et al.	Computerized cognitive training for children with neurofibromatosis type 1: a pilot resting-state fMRI study	2017
Zhang, et al.	Comparing the transfer effects of three nonpharmacological interventions in children with AD/HD: a single-case experimental design	2020

More than half of the studies (52/98) were designed as randomized controlled trials (RCTs). Therefore, they were classified as Level II, while Level IV emerged as the second largest group (25/98), including case series with either post-test or pre−/post-test outcomes. The remaining papers were assigned to the sub-classification of Level III, depending on whether they described pseudorandomized-controlled trials (Level III-1; 6/98) or comparative studies with or without concurrent controls (respectively Level III-2; 14/98 and Level III-3; 1/98). Furthermore, we verified the presence and the features of the control groups. While a subset of the included studies (29/98–30%) was designed without control groups, in most papers (69/98–70%), the subjects were compared to a group of healthy controls (5/69) or subjects undergoing treatment as usual (i.e., rehabilitative sessions not including telerehabilitation—17/69), no treatment/waitlist (21/69), placebo treatments (11/69), or same/different telerehabilitation treatment with different features (e.g., frequency and duration of the rehabilitative sessions—11/69); a small minority (4/69) of the studies were designed with more than a control group: two papers included a no-treatment/waitlist and a placebo group, while the other two included a placebo and a same/different telerehabilitation treatment group.

### 3.1 Population

**Figure 2:**
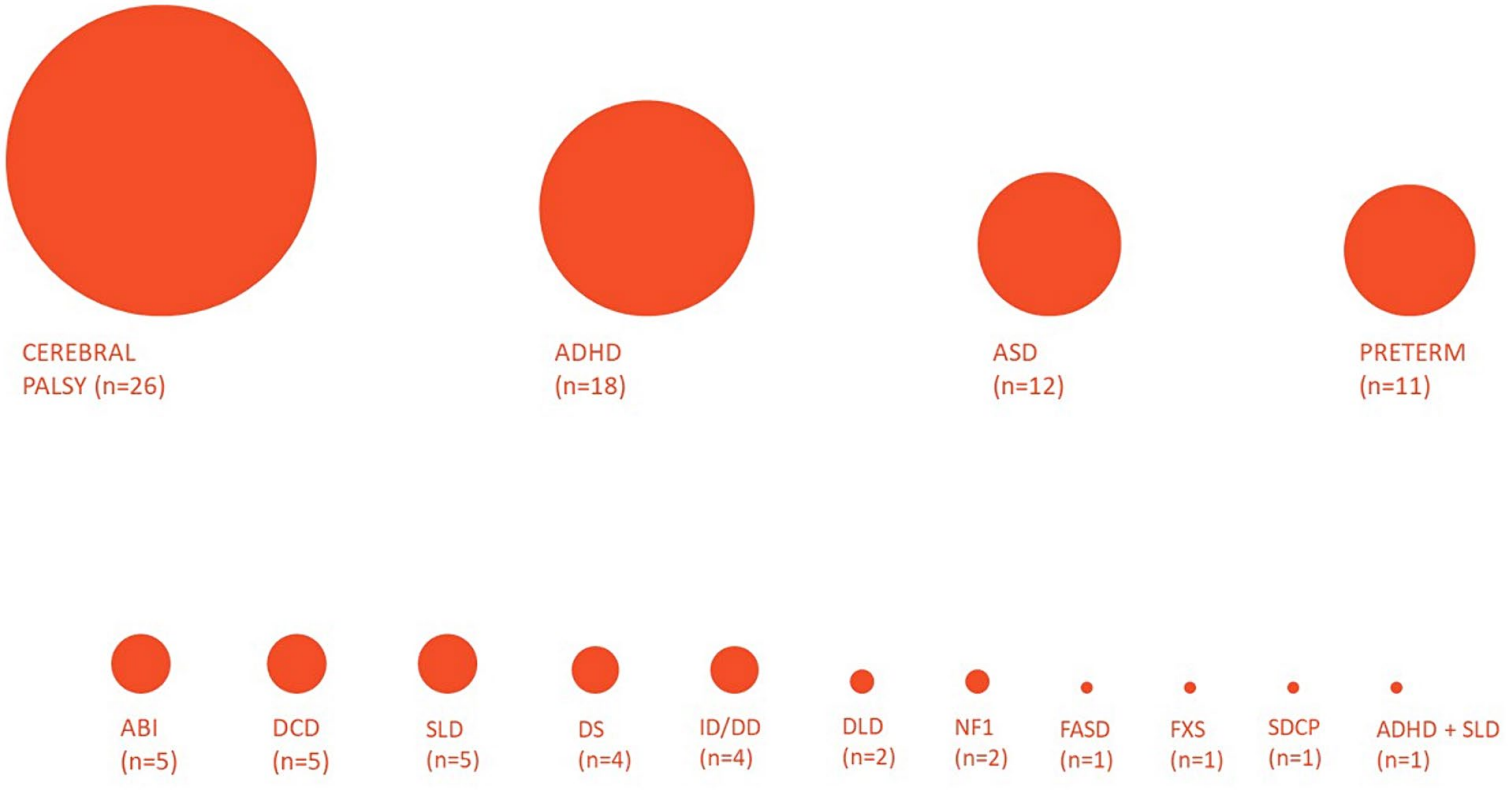
The landscape of neurological and neurodevelopmental disorders: the figure represents the distribution of the reviewed papers according to the nosographic classification of their populations. The diameter of the bubbles is proportional to the numerosity of the groups. ADHD, Attention Deficit and Hyperactivity Disorder; ASD, Autistic Spectrum Disorder; ABI, Acquired Brain Injury; DCD, Developmental Coordination Disorder; SLD, Specific Learning Disabilities; DS, Down Syndrome; ID/DD, Intellectual Disability/Developmental Delay; NF1, Type 1 Neurofibromatosis; FASD, Fetal Alcohol Spectrum Disorder; FXS, Fragile-X Syndrome; SDCP, speech disorder associated to cleft palate.

The applied population criteria also yielded a heterogeneous representation of the neuropsychiatric conditions treated via technological tools for telerehabilitation (see Figure 2). Based on the epidemiology of this nosographic group, the most numerous papers (47/98–48%) included papers describing interventions for patients with neurodevelopmental disorders. “Neurodevelopmental disorders” is an umbrella term, including various diseases with different clinical features; thus, a more specific analysis was performed: the two most represented pathologies were Attention Deficit and Hyperactivity Disorder (ADHD) and Autism Spectrum Disorder (ASD) (respectively, 18/98–18%, and 12/98–12%), followed by the Developmental Coordination Disorder (DCD) (5/98–5%), the Specific Learning Disabilities (SLD) (5/98–5%) and the Developmental Delay/Intellectual Disability (DD/ID) (4/98–4%); a few papers about Developmental Language Disorders (DLD) (2/98–2%) and a sample of patient presenting a combination of SLD and ADHD (1/98–1%) were included too. Besides neurodevelopmental disorders, two other significant subgroups emerged, including papers about technological telerehabilitation protocols in patients with cerebral palsy (26/98–27%) and preterm newborns (11/98–11%). The group of paper not classified in the previous categories consisted of a collection of other conditions, such as acquired brain lesions (5/98–5%), Down Syndrome (4/98–4%), Type 1 Neurofibromatosis (2/98–2%), Fragile-X Syndrome, fetal alcohol spectrum disorders, and speech disorders associated to cleft palate (1/98–1% each).

Such heterogeneity also emerged when the populations of the reviewed papers were analyzed in terms of age range (from 3 months to 18 years) and sample size (from 3 to 180 patients).

### 3.2 Interventions

Papers about totally clinic-based rehabilitative care were excluded from the review. Thus, the settings were analyzed based on the type of adopted ecological environment (home or school) and the direct recipient of the intervention (patient or caregiver or patient+caregiver/teacher). Almost all studies directly targeted patients (87/98–89%) in a home-based setting (88/98–90%). However, the vast majority of the included papers (89/98–91%) explicitly mentioned the role of the caregivers in the tele-rehabilitative sessions. We adopted the framework proposed in a previously published review (14) to classify the type of roles that parents assumed in the intervention, as described in the method section. More than one label could be assigned to a single paper to describe the features of the caregiver involvement completely. In most papers, the caregivers were described as the subjects having the responsibility to ensure the child’s attendance to the rehabilitative sessions, encourage/motivate them to complete the intervention, and share information (e.g., child’s behavior, family needs) with the therapists or the researchers (in detail: “Bringer” 81/98; “Supporter” 71/98; “Informer” 78/98). As this review was focused on telerehabilitation, many interventions included pre-training sessions to show and teach caregivers how to use the technological devices or conduct the rehabilitative session at home; besides, such an approach was the milestone of the interventions targeting directly caregivers (54–56, 75). Thus, a significant subset of papers was classified into the “Observer” and “Learner” categories (in detail: “Observer” 37/98; “Learner” 48/98). The “Implementer” label was applied (in detail: “Implementer” 20/98) when caregivers were reported to play an active role in the telerehabilitation activities but not for every home-based task, even if it was described as part of the intervention (e.g., we did not use this label when caregivers were merely asked to install software and supervise its use). A smaller subset of papers outlined a therapeutic relationship where professionals and caregivers share ideas to adapt the rehabilitative program (“Adaptor” 9/98) (8, 51, 55, 75, 82, 85, 93, 95, 98) or have an active dialog to set the focus of the intervention (“Collaborative Decision Maker” 1/98) (98).

**Figure 3:**
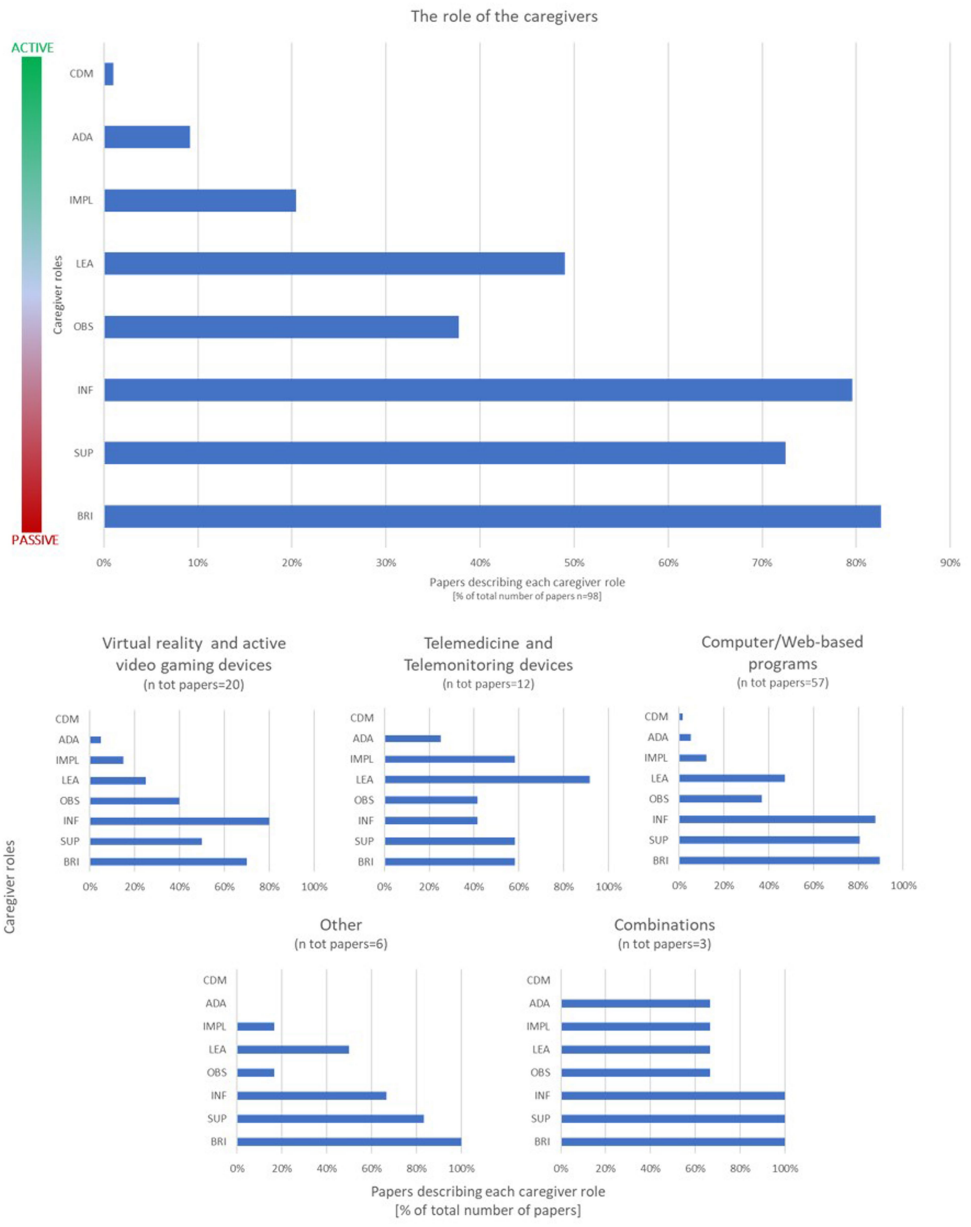
The role of the caregivers and the impact of technologies. The classification of the caregivers’ role is summarized in the bar graphs above. The upper one represents the distribution of the labels applied to the involvement of the caregivers described in the reviewed papers (more than one label could be applied to each article). The labels are reported on the axis according to the spectrum from “passive” to “active,” which is represented alongside the bar graph. The lower graphs represent the results of the cross-application of the classification of the caregivers’ role and the technologies taxonomy. The results are expressed in percentage of paper describing each role out of total number of papers included in the review (upper graph) or out of the number of papers included in each technology subgroup (lower graphs). BRI, Bringer; SUP, Supported; INF, Informer; OBS, Observer; LEA, Learner; IMPL, Implementer; ADA, Adapter; CDM, Collaborative Decision Maker.

Furtherly, we cross-applied the classification of the caregivers’ role and the taxonomy of technologies to explore the influence of the different settings on the features of the therapeutic relationship: the occurrence of the “caregivers’ role” labels across the papers describing “Virtual reality and active video gaming devices,” “Computer-based programs,” “Web-based programs,” and “other devices (e.g., purely robotic/treadmill systems, sensorized tools)” reflected the general distribution. Otherwise, the interventions based on “Telemedicine and Telemonitoring devices” or combinations of the previously mentioned technologies seemed to assign active roles to the caregivers more frequently. An overview of the analysis of the role of the caregiver is provided in Figure 3.

We also characterized the rehabilitative setting based on the role of the therapist: in 65/98 studies, the program did not require the direct intervention of the therapist to administer or monitor the intervention; more precisely, a subset of these papers (41/65) described adaptive device automatically modulating the level of difficulty of the exercise based on child’s performance, while the remaining (24/65) reported pre-determined interventions with no monitoring or adaptations needed. Otherwise, 33/98 studies described the involvement of a professional who monitored and adjusted the intervention in a synchronous (9/33) or asynchronous (24/33) setting.

The selection criteria excluded the totally “clinic-based” rehabilitative programs. Still, a sub-group of papers (7/98–7%) (28, 40, 44, 61, 85, 93, 114) describing hybrid interventions (i.e., partially administered via telerehabilitation and during “in clinic” sessions) was included in the review. The remaining papers (91/98–93%) were identified as entirely administered via telerehabilitation; a sub-classification was applied to the latter group to differentiate the home-based (82/98) from the school-based programs (9/98) (27, 62, 63, 80, 103–106, 120).

**Figure 4:**
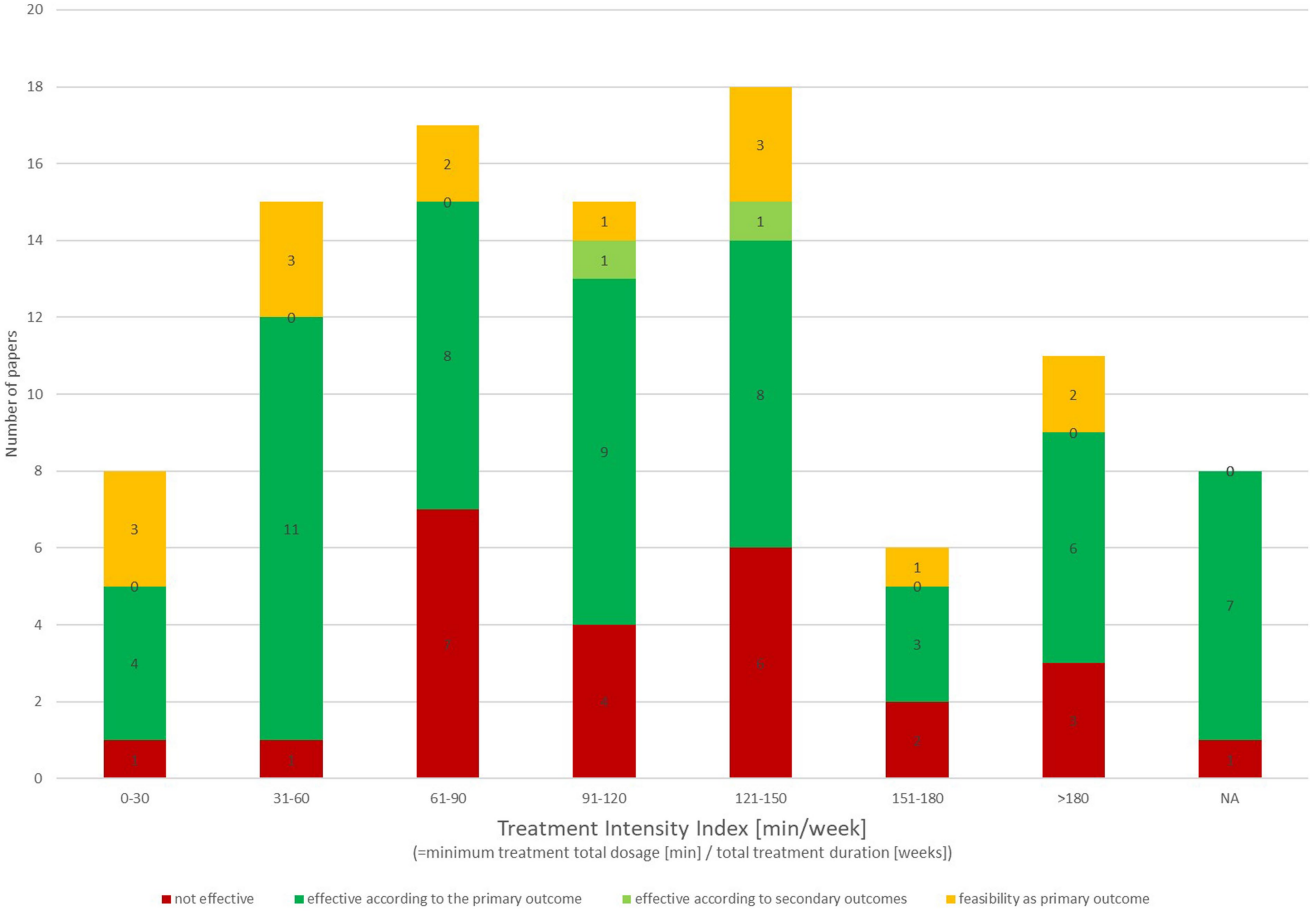
Rehabilitative workload of technological telerehabilitative interventions: the workload of the rehabilitative interventions is represented in the bar graph based on the “treatment intensity index” we applied by dividing the minimum total rehabilitative workload described in the papers (in minutes) by the total time span of the intervention (in weeks). Each bar represents a 30-min step. Bars are segmented in different colors according to the classification of effectiveness. NA, articles not containing sufficiently detailed information to calculate the index.

The workload of the rehabilitative interventions was once again largely variable, both within and between papers, in terms of frequency and duration of the sessions and total duration of the intervention. Thus, we calculated a “treatment intensity index” by dividing the minimum total rehabilitative workload described in the papers (in minutes) by the total time span of the intervention (in weeks); eight articles (8, 58, 74, 75, 77, 108, 111, 118) did not contain sufficiently detailed information to calculate the index. Such a parameter provided a comparable measure to classify the interventions’ dosage; the classification results are summarized below in Figure 4.

ICTs were analyzed using a previously published classification system to define the heterogeneous landscape of the adopted devices. The most common tools (58/98–50%) were “computer-based programs and web-based platform” (e.g., Cogmed, BrainGame Brian), followed (20/98–21%) by “virtual reality and active video-gaming” including commercially available video-gaming consoles (e.g., Nintendo Wii, Sony Playstation, Microsoft XBox) and research devices based on virtual reality. A third subset of papers (12/98–12%) analyzed rehabilitative interventions administered via “telemedicine or telemonitoring devices” (e.g., telehealth platforms, video-call platforms). A minority of studies adopted “other devices” such as research prototypes or sensorized and tele-monitored machines, and a combination of the previous categories (respectively: 6/98–6%; 3/98–3%). The outlook of the adopted ICTs and their categorization is provided in Table 3.

**Table 3:** The taxonomy of tele-rehabilitation technologies: the technological tools adopted in the reviewed studies are reported in the table and classified according to the framework we applied for the qualitative description.

Category	Rehabilitation tools
Virtual reality and active videogaming	Nintendo Wii Fit, Microsoft Xbox Kinect, VR videogame using a sensing glove, Sony PlayStation, Move and Eye motion input devices
Telemedicine or telemonitoring devices	App—phonetic training program, Zoom, video calls, video recording, RUBI-Parent Training via Telehealth, Parent Coaching Telehealth intervention
Computer-based or web-based programs	Cogmed Working Memory Training, XtraMath, Scientific Brain Training, Luminosity cognitive training, EVO platform, Braingame Brian training, Tobii X2-60, Gaming Open Library for Intervention in Autism at Home(GOLIAH), TALi Helath, Mind Reading Software, The Number Race, Focus Pocus, NeuroScouting, Reading Trainer®, The Emotion Trainer, Computer-Assisted Arm Rehabilitation (CAAR), ABRACADABRA program, “Move it to improve it” (Mitii), MoveHero, RuntheRAN, Web App “I bambini contano”
Other devices	Home-based virtual cycling training (hVCT), home-based intelligent stretching robot, MOTOMed gracile, Self-Initiated Prone Progression Crawler (SIPPC) robotic system, Google glasses+Android app, CareToy platform
Combination of the previous categories	Microsoft Xbox360 + Kinect; Sony PlayStation3 + Move and Eye input devices; Google glasses+Android app, Focus Pocus+ EEG hardware,Computer videogames + EEG (neurofeedback), Pre-recorded video clip+Kinect 3D camera,+video-connection

The rehabilitative interventions were analyzed based on the skills (neuropsychological, motor, or speech and communication abilities) they were designed to address and the type of outcome measures adopted to assess their effectiveness.

Most of the described protocols were designed to train functions of a single domain, in particular neuropsychological (e.g., cognitive skills, executive functions, academic skills) or motor (e.g., gross motor functions, balance, coordination) functions (respectively 53/98–54%; 34/98–35%). Only a small minority (3/98–3%) of the reviewed paper described rehabilitative tools aiming to train speech and communication skills specifically. Moreover, we identified a subset of papers reporting multimodal tele-rehabilitation tools that simultaneously targeted neuropsychological and motor (5/98–5%) or speech and communication (3/98–3%) skills.

### 3.3 Outcomes

Each primary outcome measure of the paper selected (274 variables in total) was classified based on the assessed function, into the four broad components of the *International Classification of Functioning, Disability and Health for Children and Youth* (ICF-CY) (26). Most of the tools adopted to assess the outcome of neuropsychological and motor rehabilitative tools fell into the “Body Functions” category, mainly because the trained skills could be classified as “global/specific mental functions” (128/169) or “movement functions” (35/169), thus this domain resulted in being the largest (164/274–59,9%%). The “Activities and Participation” domain is less represented as 91/274 (33,2%) outcome measure could be such classified, including mostly “mobility” (30/91) and “learning and applying knowledge” (44/91) chapters. No papers primarily assessing skills specifically attributable to the “Body structure” and “Environmental Factors” were identified. However, a subgroup of papers adopted a composite battery of primary outcome measures, assessing beyond parameters classifiable into the “Body Functions” or “Activities and Participation “variables into the “Body structures” domain (5/274–1,8%) categories. The remaining reviewed articles (14/274–5,1%) reported “feasibility” as the main outcome measure, therefore they were not included in this analysis.

We eventually classified the included papers based on their results (i.e., non-efficacy, efficacy based on the primary outcome/other outcomes, feasibility). Overall, 59% of the reviewed papers documented the effectiveness of the intervention based on the primary outcome (57%) or secondary outcomes (2%); the subgroup including the studies having feasibility as primary outcome was not included in the efficacy categorization.

**Figure 5:**
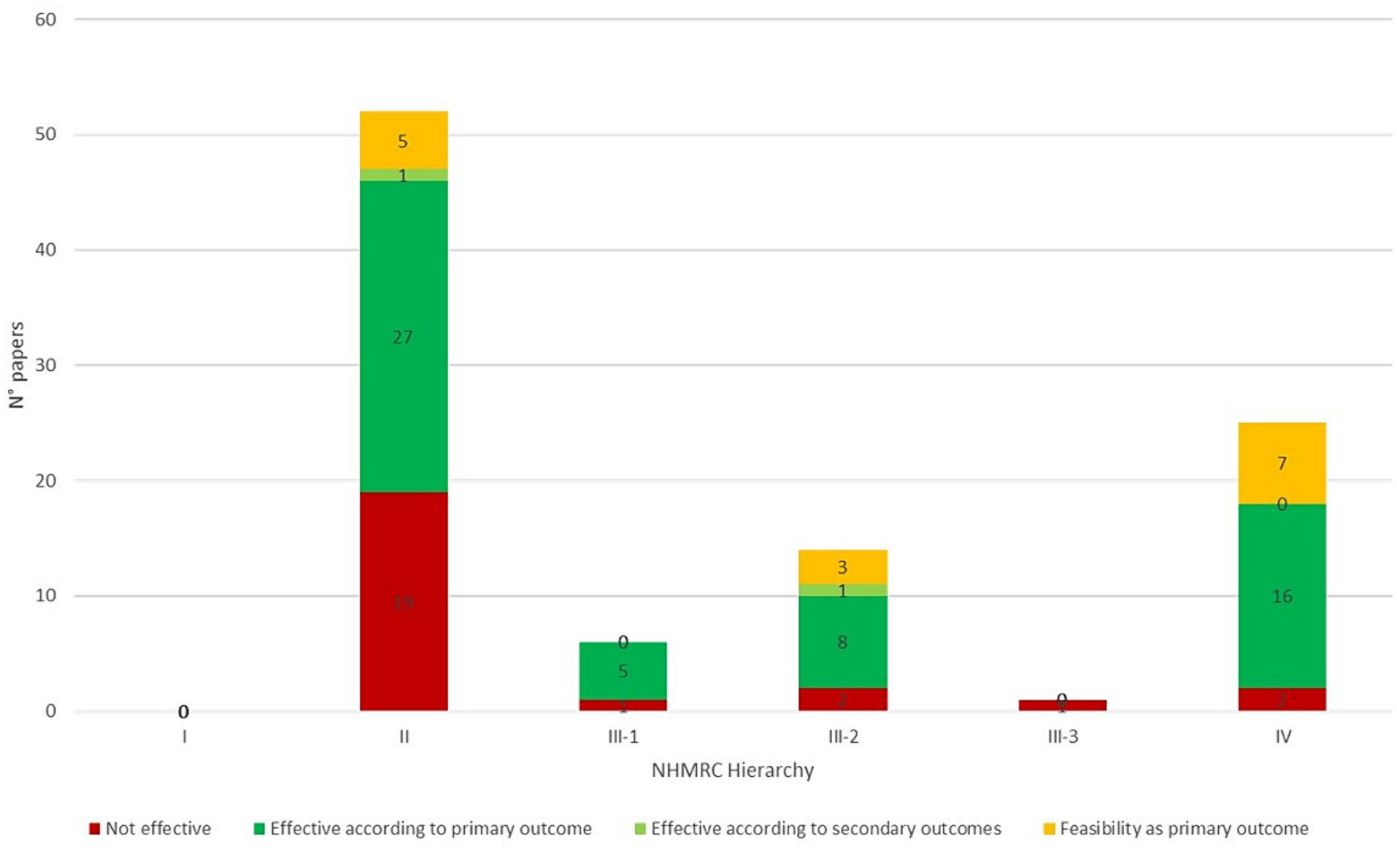
Evidence grade and effectiveness of technological telerehabilitative interventions: the bar graph summarizes the qualitative description of the evidence grade and the effectiveness of the reviewed papers. The study design was classified according to the NHMRC Hierarchy and effectiveness was labeled according to the outcomes. Bars are segmented in different colors according to the classification of effectiveness. NHMRC, National Health and Medical Research Council.

The results of this analysis, subclassified per grade of evidence and “Intensity index,” are summarized in Figures 4, 5.

## 4 Discussion

**Figure 6:**
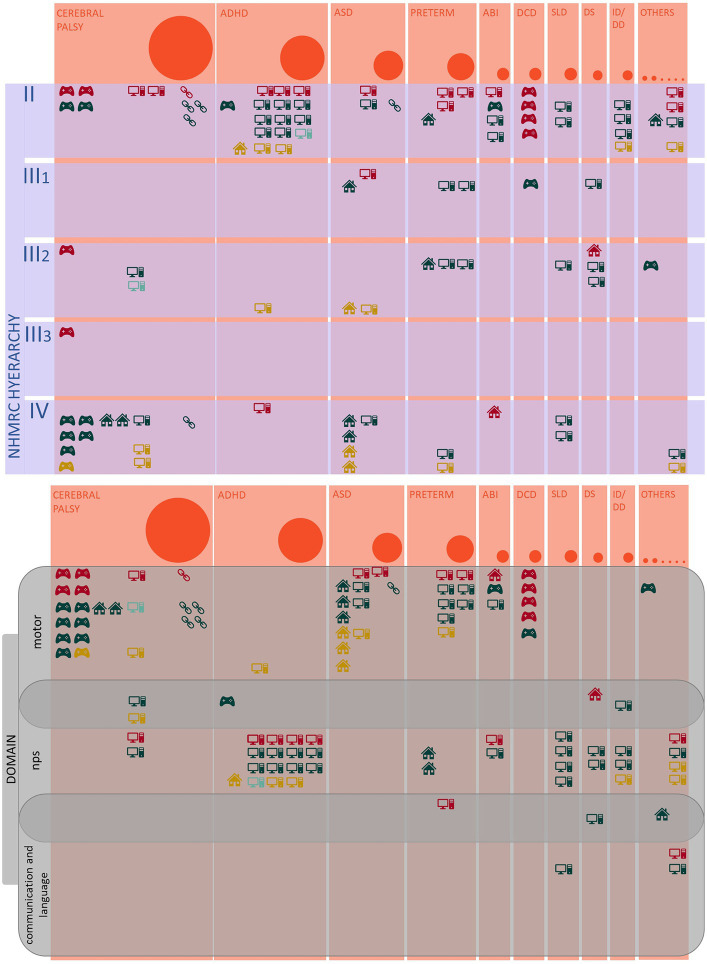
The landscape of technologic telerehabilitation for pediatric neurologic and neurodevelopmental disorders: the infographic summarizes the main analyzed variables of the reviewed papers. The bubbles’ diameter and the orange columns’ width are proportional to the number of identified papers per diagnostic group. The icons represent the classification of the adopted technological devices (see below); every icon corresponds to a single paper. The colors correspond to the classification of the efficacy of the interventions described in each paper (i.e., red, not effective; dark green, effective based on primary outcome; light green, effective based on secondary outcomes; gold, feasibility as primary outcome). 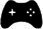, Virtual reality and active video gaming devices; 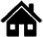, Telemedicine and Telemonitoring devices; 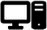 Computer-based program; 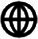, Web-based platform; 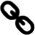, other devices.

For the purpose of this review, we adopted a wide-scope search strategy to encompass as extensively as possible the multifaceted field of technological telerehabilitation for pediatric neurologic and neurodevelopmental disorders. Consequently, the paper selection process yielded many papers composing a heterogeneous landscape (Figure 6), mainly in terms of population and study design. The two most numerous sub-groups of articles included samples of patients affected by cerebral palsy and neurodevelopmental diseases, as expected based on the epidemiology of pediatric neuropsychiatric disorders. Besides, two other recurring conditions were Acquired Brain Injuries and Preterms. At the same time, the remaining few included a group of other pathologies studied in a single or a couple of papers. Notably, the distribution of the studies about neurodevelopmental disorders is unbalanced in favor of ADHD and ASD, while other disorders with high prevalence (e.g., SLD) were less represented. Furthermore, our search did not intercept other common neuropsychiatric conditions (e.g., epilepsy, neuromuscular diseases) in the reviewed paper. This finding may be due to the features of the search string. However, it suggests that there are areas where the application of technological telerehabilitation is still to be explored.

Despite the majority of the protocols was structured as RCTs, sample sizes and the study design differed widely. The diversity in the pathogenesis of the diseases and the variability in the study design and the adopted outcome measures made it unfeasible to do a meta-analysis for comparing the results of the included studies. Nonetheless, our qualitative description yielded a prevalence of papers reporting efficacy according to the selected primary/secondary outcome measures in every NHMRC Hierarchy Class. This distribution might be influenced by publication biases. Still, it also provides preliminary support for the effectiveness of this kind of rehabilitative approach, even if it needs to be confirmed by specific meta-analysis focused on single domains of intervention or technological devices.

Our review aimed to provide a comprehensive description of the features of the telerehabilitation setting in this field, and we decided to focus on (1) the role of caregivers and professionals (2), the types of adopted technologies (3), the intensity of the interventions and (4) the functional domains identified as therapeutic target.

We characterized the role of caregiver by applying to the reviewed papers a previously published classification that described a spectrum from “passive” to “active” roles (14). Even if the direct target of the intervention was the patient himself, almost all studies explicitly mentioned the involvement of caregivers in the intervention, suggesting that the tele-rehabilitative approach for pediatric diseases intrinsically supports a therapeutic relationship between families and professionals. However, our qualitative classification showed a “pyramidal” distribution, with “passive” labels (e.g., Implementer, Supporter, Informer) being more frequently applied than the “active” ones (e.g., Adaptor, Collaborative Decision Maker). The cross-application of this classification and the technologic taxonomy gave us a more detailed insight into this finding, even if the unbalanced numerosity of the “technologies” subgroups made a statistical comparison unfeasible. The occurrence of the “caregivers’ role” labels across the papers describing “Virtual reality and active video gaming devices,” “Computer-based programs,” “Web-based programs,” and “other devices” reflected the general distribution. In contrast, the interventions based on “Telemedicine and Telemonitoring devices” or combinations of the previously mentioned technologies seemed to assign active roles to the caregivers more frequently. We also classified the other side of the therapeutic relationship, by analyzing the professionals’ role in designing, administering and modulating the interventions. Notably, most studies described programs that do not require the direct intervention of the therapist to administer or monitor the intervention.

Many factors may have influenced this finding. Firstly, computer/web-based programs and devices for virtual reality and active videogaming emerged to imply more “passive” roles, as caregivers in these interventions are mainly required to supervise and support the use of the tool by the child. As these technologies were the most frequently mentioned in the reviewed papers, the features of their setting may have twisted the general description. Secondly, a significant subset of articles described technological tools having the possibility of modulating the level of difficulty of the exercise based on the child’s performance with no professional interventions needed. The intrinsic adaptivity of the technological devices was emphasized because of their potential in providing a dynamic intervention, reducing the workload of professionals and fostering the effectiveness of the rehabilitative intervention (122). However, the usability of technologies can still be a barrier to the acceptance of the telerehabilitation approach by the families (21) and, as mentioned above, “active” caregivers’ roles imply the collaborative interaction with the therapist.

Regarding the analysis of the adopted technologies for telerehabilitation, to date a standardized taxonomy able to classify is still lacking. We integrated previously published classifications to define a novel taxonomy for digital technologies that could consider all the domains handled by clinicians. The categories we proposed encompass all devices targeting purely motor, neuropsychological or speech treatments but also integrated ones, thus, by combining motor and cognitive or cognitive and speech. Functions.

The most commonly adopted ICTs were computer-based/web-based programs and virtual reality and active video-gaming devices, while a smaller subset of papers described telemedicine/telemonitoring devices or tools combining different technologies. Some issues may be raised from this situation. As mentioned above, the computer-based/web-based and virtual reality/active video-gaming types of technologies appeared to be related to a more “passive” role of the caregiver. Besides, more advanced integrated technologies (e.g., equipped with wearable sensors or remotely monitorable) are not yet very diffused across clinical studies.

The data about the rehabilitative interventions’ workload—in terms of frequency and duration of the sessions, and total duration of the intervention—were once again largely variable, both within and between papers. The “treatment intensity index” we applied provided an approximate but comparable measure to classify the dosage of such diverse interventions. Interestingly, the majority of the interventions (70/98) included a weekly workload of 60 min or more. This finding might be due to the research setting, prioritizing shorter and more intense interventions. However, it also suggests the potentiality of the home-based setting in integrating the in-clinic session increasing the dosage of the intervention.

The description of the main features of the technological tele-rehabilitative setting was completed by the analysis of the interventions based on the skills they were designed to address, and the type of outcome measures adopted to assess their effectiveness.

Overall, a prevalence of single-domain intervention emerged, in particular focused on neuropsychological or motor functions. Interestingly, we also identified a subset of papers reporting multimodal tele-rehabilitation tools which simultaneously targeted neuropsychological and motor or speech and communication skills.

We aimed to further characterize the objectives of the interventions classifying the main outcome measures, based on the assessed function, into the four broad components of the ICF-CY. As outlined in the Results section, most of the primary outcome measures of the reviewed telerehabilitation programs could be classified in the “Body function,” according to reviews on ICF domains mainly targeted by interventions (34), even though family and child goals tend to be focused on activities and participation. It is therefore of utmost importance to conceptualize technological treatment pathways that conceive both the improvement of function and quality of life integrated as primary goals and targets of the intervention.

To the best of our knowledge, this is the first systematically conducted review providing a wide-scope overview of the heterogeneous landscape of technological telerehabilitation for pediatric neurologic and neurodevelopmental disorders. Our results provide a detailed qualitative description that can be a base for planning future policies and research, considering the promising results in terms of effectiveness of telerehabilitation protocols. In particular, the following issues should be addressed based on the features emerged from this review (1): the description of a relatively “passive” caregiver role across the studies advocate for a further exploitation of the potentials of the technological telerehabilitation approach as a setting where caregivers and professionals can cooperate in an actual active family-centered care (2); the creation of a standardized classification shared by the different professional figures involved in this field (e.g., by a consensus panel) is needed to improve clinical practice, scientific research, and comparative work (3); given the vast heterogeneity of the interventions, the efficacy of this approach needs to be confirmed by specific meta-analysis focused on comparable domains of interventions or technological devices (4); the potential of adopting advanced technologies and multidomain interventions should be further explored, to address the clinical needs of the most common pediatric neurological and neurodevelopmental diseases often including complex and multifaceted impairments.

## Data availability statement

Authors confirm that all data generated and analyzed during this study are included in the manuscript and they are available in Zenodo Repository (https://zenodo.org/doi/10.5281/zenodo.10820040) upon request to the corresponding author.

## Author contributions

BL: Conceptualization, Data curation, Methodology, Writing – original draft, Writing – review & editing. SP: Data curation, Methodology, Writing – original draft, Writing – review & editing, Visualization. SF: Data curation, Methodology, Writing – original draft, Writing – review & editing. GM: Writing – review & editing, Investigation. EB: Writing – review & editing, Methodology. ML: Writing – review & editing, Methodology. AB: Investigation, Writing – review & editing. MB: Investigation, Writing – review & editing. MP: Investigation, Writing – review & editing. CT: Investigation, Writing – review & editing. FF: Writing – review & editing. GC: Writing – review & editing. GS: Writing – review & editing.

## Group member of the Italian Neuroscience and Neurorehabilitation Network

Arnoldi Maria Teresa, Baglio Francesca, Barzacchi Veronica, Bassi Maria Teresa, Berardinelli Angela, Bombonato Clara, Borgatti Renato, Calabrò Rocco Salvatore, Cardillo Ilaria, Castelli Enrico, Cavallini Anna, Ceragioli Beatrice, Cersosimo Antonella, Condoluci Claudia, Corti Claudia, Di Girolamo Gabriella, Di Giusto Valentina, Elia Maurizio, Favetta Martina, Ferrante Carolina, Ferri Raffaele, Ghione Valeria, Goffredo Michela, Lugari Patrizia, Manzia Carlotta Maria, Martini Giada, Matteucci Elisa, Menici Valentina, Moretti Paolo, Pagliano Emanuela, Perinelli Martina Giorgia, Petrarca Maurizio, Poggi Geraldina, Pulvirenti Francesca, Rizzo Marta, Sgherri Giada, Strazzer Sandra, Striano Pasquale, Tassorelli Cristina, Vannetti Federica, Viganò Marta.
